# The *Phaseolus vulgaris* ZIP gene family: identification, characterization, mapping, and gene expression

**DOI:** 10.3389/fpls.2013.00286

**Published:** 2013-07-30

**Authors:** Carolina Astudillo, Andrea C. Fernandez, Matthew W. Blair, Karen A. Cichy

**Affiliations:** ^1^Plant Soil and Microbial Sciences Department, Michigan State UniversityEast Lansing, MI, USA; ^2^Department of Plant Breeding and Genetics, Cornell UniversityIthaca, NY, USA; ^3^Plant Soil and Microbial Sciences Department, USDA-ARS Sugarbeet and Bean Research Unit, Michigan State UniversityEast Lansing, MI, USA

**Keywords:** biofortification, *Phaseolus vulgaris*, QTL mapping, gene expression, zinc transporter

## Abstract

Zinc is an essential mineral for humans and plants and is involved in many physiological and biochemical processes. In humans, Zn deficiency has been associated with retarded growth and reduction of immune response. In plants, Zn is an essential component of more than 300 enzymes including RNA polymerase, alkaline phosphatase, alcohol dehydrogenase, Cu/Zn superoxidase dismutase, and carbonic anhydrase. The accumulation of Zn in plants involves many genes and characterization of the role of these genes will be useful in biofortification. Here we report the identification and phlyogenetic and sequence characterization of the 23 members of the ZIP (ZRT, IRT like protein) family of metal transporters and three transcription factors of the bZIP family in *Phaseolus vulgaris* L. Expression patterns of seven of these genes were characterized in two bean genotypes (G19833 and DOR364) under two Zn treatments. Tissue analyzed included roots and leaves at vegetative and flowering stages, and pods at 20 days after flowering. Four of the genes, *PvZIP12, PvZIP13, PvZIP16*, and *Pv bZIP1*, showed differential expression based on tissue, Zn treatment, and/or genotype. *PvZIP12* and *PvZIP13* were both more highly expressed in G19833 than DOR364. *PvZIP12* was most highly expressed in vegetative leaves under the Zn (−) treatment. *PvZIP16* was highly expressed in leaf tissue, especially leaf tissue at flowering stage grown in the Zn (−) treatment. *Pv bZIP1* was most highly expressed in leaf and pod tissue. The 23 *Pv*ZIP genes and three bZIP genes were mapped on the DOR364 × G19833 linkage map. *PvZIP12, PvZIP13*, and *PvZIP18, Pv bZIP2*, and *Pv bZIP3* were located near QTLs for Zn accumulation in the seed. Based on the expression and mapping results, *PvZIP12* is a good candidate gene for increasing seed Zn concentration and increase understanding of the role of ZIP genes in metal uptake, distribution, and accumulation of zinc in *P. vulgaris*.

## Introduction

Dry beans (*Phaseolus vulgaris* L) are the most highly consumed whole food legume in the world. Beans are a food security crop for small farmers and urban poor in many African and Latin American countries (Siddiq and Uebersax, 2012). In contrast to many other staple crops, beans are rich in a variety of nutrients, including protein, fiber, folate, and minerals (Juliano, 1999). Beans are also a good source of dietary iron and zinc. According to the USDA Nutrient Database, a 100 g of cooked beans provides an average of 2 mg Fe and 1 mg Zn and the Estimated Average Requirement for Fe ranges from 3 to 23 mg per day and 2.5–10.9 mg per day per Zn depending on age and gender (Ahuja et al., 2012). Meeting the Fe and Zn dietary requirements is a challenge for many people. An estimated two billion people suffer from iron deficiency, which is a major cause of anemia (Rastogi and Mathers, 2002; Balarajan et al., 2011). Zinc deficiency is also widespread, with an estimated 48% of humans at risk, especially populations consuming vegetarian diets rich in unrefined cereals (Sandstead, 1991). In humans, Zn deficiency can be expressed through diverse symptoms including reduced immune function, fetal brain cell development and child's growth, reproductive, and cognitive development (Hambidge, 2000). Biofortification of staple foods, including dry beans, with Fe and Zn is one agricultural based approach being developed and applied to combat micronutrient malnutrition (Bouis et al., 2011). While average dry bean Fe and Zn levels are 55 mg kg^−1^ and 34 mg kg^−1^ respectively, three-fold genotypic variation in both Fe and Zn levels exist within the species (Blair et al., 2009 and Islam et al., 2002).

This existing variation makes breeding common beans a viable biofortification approach. Significant progress has been achieved in Fe biofortification of beans through conventional breeding as illustrated in the recent release of five high Fe bean varieties in Rwanda (Saltzman et al., 2013). Zinc biofortification has lagged behind that of Fe-biofortification perhaps because of lower quantities of Zn in the seeds but also perhaps less incentive because of the difficulty in assessing Zn nutritional status in humans. While there are biomarkers to asses Fe deficiency readily in humans, no such biomarkers are yet available for Zn, although recently a potential biomarker (dematin) has been identified (Ryu et al., 2012).

In addition to relying solely on phenotypic selection to increase seed Fe and Zn levels, there has been an effort to understand the genetic control of seed Zn and Fe accumulation. Since 2009, at least five QTL studies have been published for seed micronutrient levels. In total, 38 QTLs were associated with zinc accumulation, explaining 15–40% of the variability. These studies have been in inter gene pool populations (Blair et al., 2009, 2010a,b), Andean populations (Cichy et al., 2009; Blair et al., 2011) and Mesoamerican populations (Blair et al., 2010a). QTL studies have yet to be applied to marker assisted selection. There has also been limited effort in identifying genes underlying QTL for Fe and Zn. Discovery of genes involved in increased seed Fe and Zn levels would be useful for biofortification efforts in beans and possibly also as targets for transgenic biofortification approach in other crops.

The Zrt and Irt-like protein (ZIP) family is well characterized for its role in Zn transport and to a lesser extent it role in Fe transport (Eide et al., 1996). The ZIP family is well conserved among bacteria, fungi, protozoa, animals, and plants (Grotz et al., 1998; Chen et al., 2008). ZIP proteins are predicted to have eight trans-membrane domains with a histidine motif which may be part of an intramembranous heavy metal binding site that plays a role in the transport pathway for the minerals that are transferred (Eng et al., 1998). ZIP transporters have been implicated in Zn uptake, transport of Zn in leaves and translocation to seeds, embryo, endosperm, and seed coat (Waters and Sankaran, 2011). Previous information on the role of ZIP genes in Zn movement throughout the plant come from expression analysis, yeast complementation and Zn hyper accumulator mutants. In *A. thaliana* 15 members have been identified and characterized, revealing a wide variety of localization and function (Milner et al., 2012). *At*ZIPs have been detected mainly in the roots, shoots (Milner et al., 2012). In rice, 17 ZIP coding sequences (CDS) were identified. They have been evaluated in roots, shoots, and panicles of efficient and inefficient genotypes (Grotz et al., 1998; Guerinot, 2000; Connolly et al., 2002; Weber et al., 2004; Chen et al., 2008; Shanmugam et al., 2011; Milner et al., 2012). In *Medicago truncatula*, six genes were identified in roots and leaves which were upregulated under Zn deficiency and three of them restored yeast growth on Zn-limited media (Lopez-Millan et al., 2004). In *Glycine max, GmZIP1* has been detected in nodules and was highly selective for Zn in a functional complement in yeast (Moreau et al., 2001). In *Vitis vinifera, VvZIP3* was expressed in developing flowers and its expression was correlated with high Zn accumulation in this tissue (Gainza-Cortes et al., 2012 and Afoufa-Bastien et al., 2010). Analysis of this family in different species demonstrates the importance of these genes in Zn transport.

Another important gene family related with Zn transport is the bZIP family. This family has been well characterized in *Arabidopsis* with 75 members divided in 10 groups based on conserved motifs that reflect functional similarities (Jakoby et al., 2002). Group F includes bZIP19, bZIP23, and bZIP24. These transcription factors contain a DNA binding domain, a leucine zipper dimerization motif and histidine-rich motif which are essential for responding to low Zn supply in *Arabidopsis* (Assuncao et al., 2003; Assunção et al., 2010).

With the recent release of the *P. vulgaris* genome sequence (*Phaseolus vulgaris* v1.0, DOE-JGI and USDA-NIFA, http://www.phytozome.net/commonbean), it is possible to identify candidate genes for seed Fe and Zn levels. Characterization of genes related to Zn homeostasis in *P. vulgaris* will provide useful information on specific target genes in the biofortification breeding effort. This research has identified and characterized of 23 members of the *Pv*ZIP gene family. Three members of a second family of genes, bZIP transcription factors, were also characterized similarly. The relative expression of genes from both the ZIP and bZIP families was characterized in various tissues and stages of development in two *P. vulgaris* genotypes, DOR 364 and G19833 grown under two Zn treatments. Selected ZIP and bZIP genes were also located on a linkage map overlaid with QTL locations for Zn accumulation in seed.

## Materials and methods

### Plant material and phenotypic data

Two bean genotypes were evaluated in this study, DOR364, a small seeded, high yielding improved cultivar from the Middle American genepool and G19833, a large seeded landrace from the Andean genepool known for its tolerance to low P soils (Beebe et al., 2006). These genotypes also exhibit contrasting seed mineral levels as shown in field trials in Darien, Colombia. DOR364 had 49 mg kg^−1^ Fe while G19833 had 75.5 mg kg^−1^, and DOR364 had 21.7 mg kg^−1^ Zn while G19833 had 29.9 mg kg^−1^ (Blair et al., 2009). DOR364 and G19833 were specifically chosen for this study because valuable genetic information exists for the lines. A recombinant inbred line (RIL) between these parents was developed by single seed descent at the International Center for Tropical Agriculture (CIAT), Colombia. It consists of 87 individuals and has a linkage map of 499 single copy markers with a coverage of 2306 cM (Galeano et al., 2011). This population has been used by different research groups for map saturation and QTL identification associated to biotic and abiotic traits (Blair et al., 2009) and QTL positions for seed minerals (Blair et al., 2003, 2009; Beebe et al., 2006; Galeano et al., 2011).

### Identification of *Pv* ZIP and *Pv* bZIP genes and phylogenetic analysis

ZIP genes in *P. vulgaris* were identified using the sequences of 18 *Arabidopsis thaliana* ZIP genes (http://www.arabidopsis.org/). The program tBlastn was used to compare the *Arabidopsis* ZIP genes against the bean genome (*Phaseolus vulgaris* v1.0, DOE-JGI and USDA-NIFA, http://www.phytozome.net/commonbean). These sequence data were produced by the US Department of Energy Joint Genome Institute. Conserved domains in each predicted transcript was verified using Pfam 26.0 protein database (http://pfam.sanger.ac.uk/) to confirm the reliability of the match with the ZIP family. The CDS for each gene was aligned with genomic DNA sequence to confirm splice signals in boundaries between introns and exons. The *P. vulgaris* ZIP genes were assigned unique names from *PvZIP1* to *PvZIP19* and *PvIRT1 to PvIRT4*. These names do not relate to naming of ZIP genes in others species. Since this gene family characterization is based on an incomplete genome sequence, the existence of additional ZIP genes in the bean genome is a possibility.

Three *Pv* bZIP genes were identified in the *P. vulgaris* genome based on sequences of *bZIP19, bZIP23*, and *bZIP24* reported by Assunção et al. (2010) in *Arabidopsis*. Identification of the new bZIP genes was based on the homology with the Basic Leucine Zipper Domain (bZIP domain).

Sequence alignments, phylogenetic analysis, tree estimation using bootstrapping and graphs of each gene were performed using ClustalW (Larkin et al., 2007) using the program Geneious® 6.0.3, created by Biomatters (build 2012-11-06 10:52).

### *in silico* mapping of *Pv* ZIP and *Pv* bZIP genes

Each of the 23 putative ZIP transport protein genes and 3 putative bZIP transcription factor genes were mapped *in silico* to a location on the DOR364 x G19833 linkage map based on sequence homology with the *P. vulgaris* genome. This alignment was conducted with an MS Excel based program MapSynteny (Fernandez et al., 2011).

### Genetic mapping of select members of the PvZIP and Pv bZIP family genes

Five ZIP genes were also mapped genetically in the DOR364 × G19833 population. These five genes were chosen for genetic mapping based on their location near QTLs for seed Fe and Zn concentration on chromosomes 1, 3, 6, and 8 (Blair et al., 2009). The ZIP genes located *in silico* in these regions were mapped genetically in the full set of RILs of the DOR364 × G19833 population. These include *PvZIP2, PvZIP6, PvZIP8, PvZIP13*, and *PvIRT3*. Primers were designed to flank ZIP gene intron sequence (Table 1). PCR was conducted on DOR364 and G19833 as a first step to test for polymorphisms. The PCR mix contained 2.0 mM Mg, 0.2 μM dNTPs, and 0.3 μM of each primer. PCR reactions were carried out for 3 min at 95°C, followed by 35 cycles of 30 s at 95°C, 30 s at 55 or 60°C (based on the annealing temperature of each primer), and a final period of 5 min at 72°C. Products were visualized on agarose gels to verify amplification and identify insertion/deletions that had potential to serve as molecular markers. To increase the possibility of finding polymorphisms for monomorphic products, the SSCP technique (from single strand conformational polymorphism) was used, which is based on detection of conformational differences of single stranded DNA fragments due mobility shifts in non-denaturing polyacrylamide gel electrophoresis (Orita et al., 1989) such as MDE acrylamide gels (MDE Gel Solution 250ML Lonza NJ, USA) as described in Galeano et al. (2009). For genetic mapping, Mapdisto software version 1.7 Beta 132 (Lorieux, 2012) was used to locate the position of the ZIP genes on the DOR364 × G19833 genetic map reported by Galeano et al. (2011). The command *place locus* was used to located the ZIP genes, using as criteria the highest LOD value and lowest recombination rate. The position of each ZIP gene was confirmed using the *Ripple order* command.

**Table 1:** **Primer list for gene expression analysis via RT-qPCR and genetic mapping**.

**Gene**	**Sequence**		**Approach**
PvZIP12	GGGCAGAGGCAAGTGCAGGG	GGGCGTGATGGAGATGCAGGA	RT qPCR
PvZIP13	CGCGCTCTTCGATTGCCAGGT	CCACCGGCGTGTAGTGCGTA	RT qPCR
PvZIP13	GCGGTGGCTCGTTGAGTATT	TGCTATGAGGTCAACAAGAGCC	Mapping
PvZIP16	TGCACGGTTGATGGCGACGG	ACGGAACTCCTTCGCCATCGT	RT qPCR
PvIRT3	AGAATAACACCATCCCCAAAATTA	AGTCACTATGGGAATGTCACAGAA	RT qPCR
PvIRT3	AATGCACATCGTGGGGATGC	GGCTTTAAACTGCGCTTGGG	Mapping
bZIP1	ATGCAACCCACCTGGCCCTGATGCT	TGCCTGCCCTTGTAGTTTCCTCGCT	RT qPCR
bZIP2	ATCGGGAGAAGAAGAAGGCTCGCGC	TCCGGCCCCTTATGTCCACCAGCAA	RT qPCR
bZIP3	GCAGCAGTTCTTGAGCGTGGAGGCT	TGAAGGTGGTGTTGCCGAAACCTGCA	RT qPCR
PvactinII	TGCCATCCAGGCCGTTCTTTCA	GGGGACTGTGTGGCTGACACC	RT qPCR

### QTL data and analysis

Phenotypic data for seed Fe and Zn concentration from Popayan and Darien Colombia in 1998 and 2003 were reported for this population in Blair et al. (2009). Additionally, seed Fe and Zn concentration from the same locations in 2006 (not previously reported) were used for QTL analysis with the linkage map reported in Galeano et al. (2011). QTL cartographer v. 2.5 (Wang et al., 2012) was used to find QTLs following the same parameters described in Blair et al. (2009).

### Expression analysis of select *Pv ZIP* and *Pv bZIP*

#### Plant growing conditions

Seeds of DOR364 and G19833 were surface sterilized and planted in 500 ml clay pots with 3:1 Sunshine Brand premium grade vermiculite (Sunshine Brand, TX, USA) and horticultural grade perlite (Industries, Inc., MA, USA). Half strength Hoagland solution (3 mM KNO_3_, 2 mM Ca (NO_3_)_2_ × 4H_2_O, sequestrene DTPA 10% Fe, 1.0 mM MgSO_4_ × 7H_2_O, 23.1 mM H3BO3, 0.38 mM ZnSO4 × 7H2O, 0.16 mM CuSO4 × 5H2O, 4.6 mM MoO4 × 2H2O, 1M KH2PO4 (pH to 6.0) was applied to pots a rate of 400 ml three times per week. Two Hoagland solution treatments were employed: (1) Zn (+) where Zn was added as ZnSO_4_ × 7H_2_O and (2) Zn (−). A total of three pots per genotype were planted and each one was designated as a biological replicate. The experiment was arranged as a randomized complete block design. Plants were grown in a growth chamber (1.86 m^2^) with a photoperiod of 16 h light and 8 h dark and an average of temperature of 29°C/20°C (day/night). For the vegetative samples, roots, and leaves were collected from the vegetative 3 stage (V3), when the third trifoliate leaf was unfolded at node 5. Leaf and root samples collected at flowering were harvested at the R2 stage when 30% of the flowers were opened. Pod samples were collected at 20 days after flowering. Plant tissue was collected in labeled sterilized tubes of 50 ml in liquid nitrogen and stored at −80°C.

### RNA extraction and real-time quantitative PCR

About 2 g of tissue from each sample collected was ground in liquid N2. Total RNA from root and leaf tissue of two developmental stages was extracted by RNeasy Plant Mini Kit (Qiagen). Pods were extracted following a protocol optimized for high starch samples (Li and Trick, 2005). Total RNA was stored in aliquots at −80°C. The concentration of RNA was quantified through Quant-iT™ RiboGreen (Invitrogen). Two μg of RNA of each sample were treated with DNase I and purified by 0.1 vol of 3M sodium acetate (pH 5.2) and 3 vol of 100% ethanol. cDNA synthesis was carried out by High Capacity cDNA Reverse Transcription Kits (Applied Biosystem), using 1 μg of RNA. cDNA concentration was measured by Quant-iT™ PicoGreen (Invitrogen).

The relative expression levels of eighth ZIP genes; *PvZIP2, PvZIP7, PvZIP6, PvZIP12, PvZIP13, PvZIP16, PvZIP18, and PvIRT3* and three transcription factors belonging to the bZIP family, *bZIP1, bZIP2, bZIP3* were measured using RT-qPCR. Primers for RT-qPCR were designed for each gene in such a way that they spanned one or two exons in genes with intronic regions to detect genomic DNA contamination (Table 1). Quantification of all transcripts was performed using the SuperScript III Platinum SYBR Green One-Step qRT-PCR Kit (Invitrogen, Carlsbad, CA) according to the manufacturer's instructions. In total three technical replicates of 50 ng of cDNA for each biological replicate of all tissues were used as template. Ten-fold serial dilutions were used to determine the efficiencies of each primer. RT-qPCR master-mix was prepared as follows: 1 μl of diluted cDNA, 5 μl of 2X SYBR Green Reaction Mix, 0.5 μl 3 pmol of each primer and nuclease-free water in a final volume of 10 μl. The StepOnePlus^™^ Real-Time PCR System (Applied Biosystems) was used for amplification and fluorescence measurement of each transcript at each temperature step and cycle during the reaction. Thermal cycling conditions consisted of 10 min at 95°C followed by 40 cycles of 15 s at 95°C and 45 s at 60°C. The identity and purity of the amplified product was checked through analysis of the melting curve carried out at the end of amplification. Relative gene expression was calculated using the comparative CT method (Livak and Schmittgen, 2001). bActin was used as a reference gene and root in vegetative stage Zn (−) treatment as a calibrator (Wen et al., 2005). Fold changes of greater than 2 was used as criteria to determine if genes were differentially expressed. Statistical analysis was performed using SAS V 9.3 (SAS Institute Inc., NC, USA). A repeated measurement analysis (Proc Mixed) was performed. Main effects were tested by ANOVA and a probability of *P* < 0.05 was chosen as the level of significance for the statistical test.

### Quantification of Zn concentrations in tissue

Plant tissue from two biological replicates of DOR364 and G19833 under two zinc treatments was quantified for Zn concentration. Tissue was freeze dried and ground to powder using a Geno Grinder 2000 (Spex CertiPrep, Metuchen, NJ) and zircon grinding balls. Two grams were sent to A&L Great Lakes Labs, Inc. Fort Wayne, IN, for mineral analysis by induced coupled plasma spectroscopy.

## Results

### Identification of ZIP family members and comparison with homologs in other species

**Figure 1:**
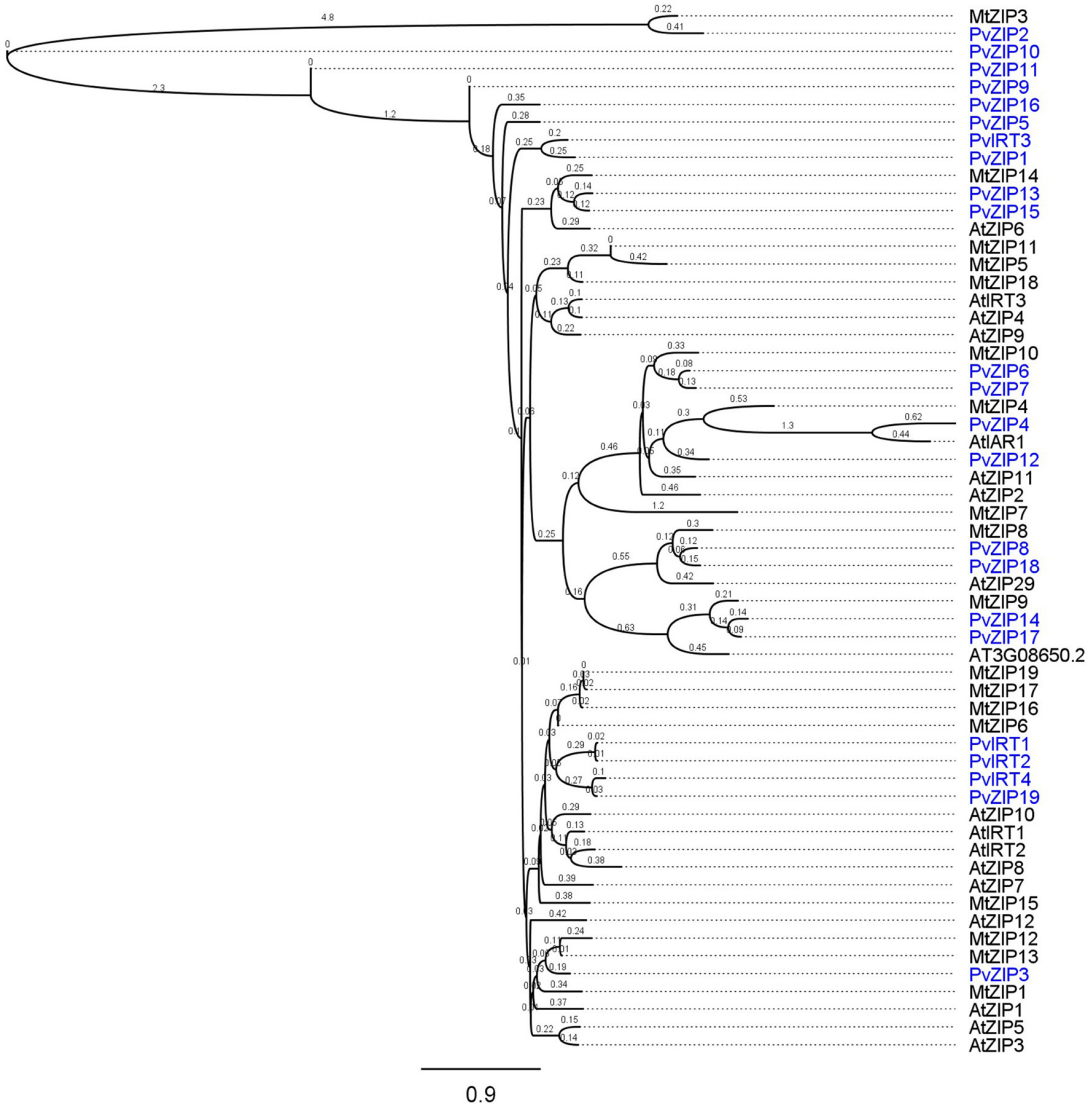
**Phylogenetic tree of homologs ZRT, IRT-like protein family in *Phaseolus vulgaris, Arabidopsis thaliana*, and *Medicago truncatula***. Analysis was based on alignment of amino acid sequences using Geneious program v. 6.0.3 and N-J trees were generated. Arabidopsis genes are indicated with the ZIP and IRT number used on TAIR database. ZIP names used in Medicago were according to Lopez-Millan et al. (2004) (ZIP1 to ZIP7). ZIP8 in front were assigned with a consecutive number.

**Figure 2:**
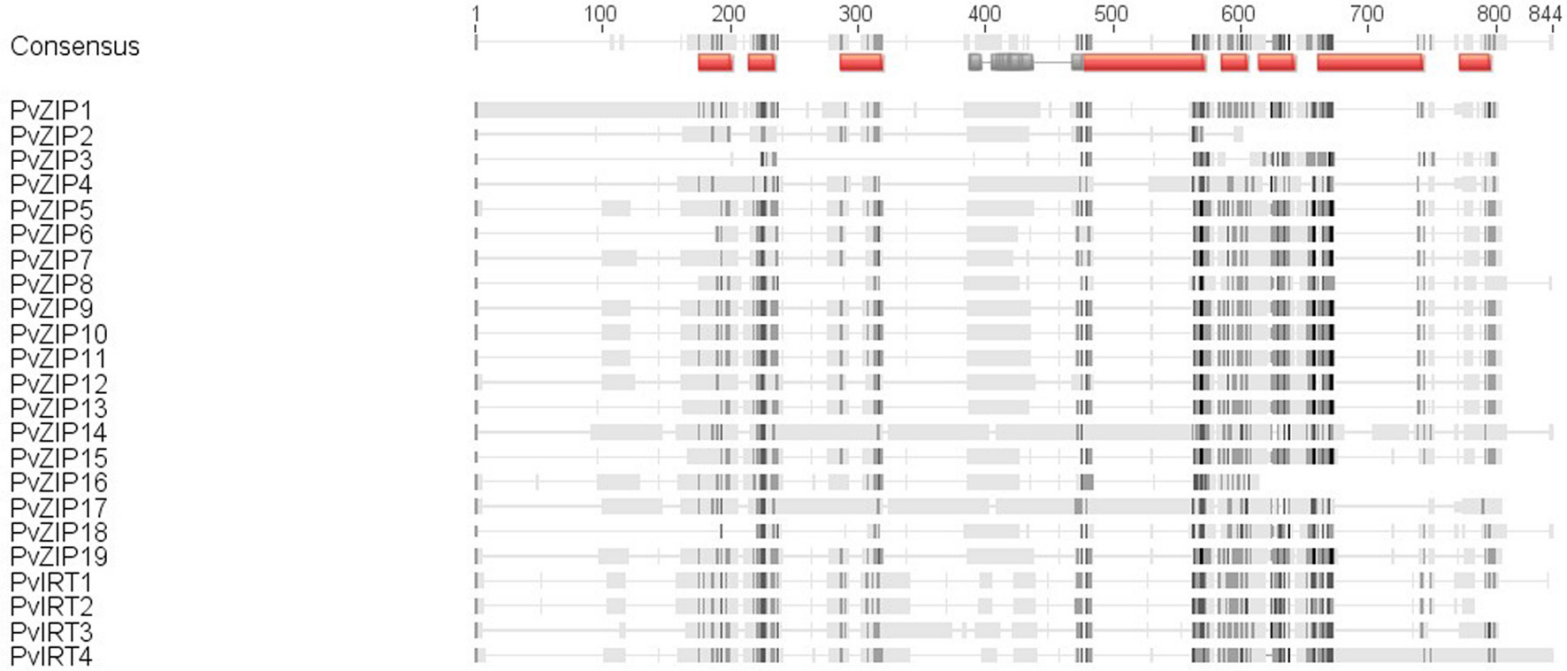
**Alignment of the predicted ZRT, IRT-like protein using CLUSTAL W**. Identical amino acids are indicated with dark shading and similar amino acids are indicated with light shading. The histidine-rich sequence located in the variable region between transmembrane domains III and IV and fully conserved histidine motifs are indicated by gray lines. The eight domains are shown as a red line above the sequences.

Twenty three sequences, including 19 ZIP and four IRT genes were identified in the *P. vulgaris* genome sequence based on similarity to ZIP genes in *A. thaliana* and/or *Medicago truncatula.* All new genes have full-length CDS containing open reading frames (ORF) ranging from 153 to 655 amino acids in length. Sequences identified were confirmed in the PFAM database based on ZIP transmembrane domain and had E-values higher than −10. Peptide sequences of all new ZIP genes identified in common bean were aligned with 18 ZIP genes reported in the *A. thaliana*, and *M. truncatula* (Table 2). A phylogenetic neighbor joining tree shows the relationship among ZIP genes in *P. vulgaris, A. thaliana*, and *M. truncatula* (Figure 1). Alignments at the amino acid level predicted eight highly conserved transmembrane domains (Figure 2) and a potential metal binding motif containing histidine residues implicated in metal transport which are highly conserved throughout the entire family (Guerinot, 2000; Lopez-Millan et al., 2004). All ZIP genes contained a histidine motif between transmembrane domain III and IV except *PvZIP6, PvZIP7*, and *PvZIP18*. The ZIP gene family members in *P. vulgaris* shared 3–81.4% homology to each other. Of all ZIP genes found *PvIRT3* was the most closely related to *Arabidopsis*, sharing 59 and 57.3% similarity with genes AtIRT3_AT1G60960.1 and AtZIP4_AT1G10970, respectively. *PvZIP14* also showed high similarity with AtZIP6_AT3G30080.1 at 53.8%.

**Table 2:** **The Zrt and Irt -like protein (ZIP) family genes and bZIP genes identified in the *P. vulgaris* genome**.

**Sequence ID**	**Gene**	**Chrom**	**Pos ition**	**Homology to *A. thaliana***		***Homology to M. truncatula***	
Phvulv091010812m	PvZIP1	Chr01	3,442,406	ATZIP4_Zinc transporter 4	3E-63	ZIP-like zinc transporter -Medtr1g016120.1	4E-145
Phvulv091015745m	PvZIP2	Chr01	49,770,995	ATZIP4_Zinc transporter 4	0	Zinc transporter 5 -Medtr3g082050.1	4E-21
Phvulv091015614m	PvZIP3	Chr01	49,839,431	ZIP3_Zinc transporter 3	4E-37	Zinc transporter 5 -Medtr3g082050.4	2E-22
Phvulv091019402m	PvZIP4	Chr02	33,735,220	IAR1_ZIP metal ion transporter	9.5E-42	Zinc transporter 5	
Phvulv091012034m	PvZIP5	Chr05	5,645,010	ZIP1_Zinc transporter 1	3E-156	Zinc transporter - Medtr3g082050.3	2E-109
Phvulv091029608m	PvZIP6	Chr05	37,426,497	ZIP2_ZRT/IRT-like protein 2	2.1E-46	Iron regulated transporter -Medtr2g097580.1	5E-99
Phvulv091029689m	PvZIP7	Chr05	37,431,839	ZIP2_ZRT/IRT-like protein 2	2E-43	Iron regulated transporter -Medtr2g097580.1	2E-161
Phvulv091029664m	PvZIP8	Chr05	37,715,863	ZTP29_ZIP metal ion transporter	6.5E-23	Zinc transporter 5 -Medtr4g065640.1	2E-130
Phvulv091026664m	PvZIP9	Chr06	200,959	ZIP5_Zinc transporter 5	1E-132	Zinc transporter -Medtr3g082050.1	1E-150
Phvulv091009317m	PvZIP10	Chr06	1,033,953	ZIP5_Zinc transporter 5	4E-130	Zinc transporter -Medtr3g082050.1	4E-44
Phvulv091009315m	PvZIP11	Chr06	1,040,964	ZIP5_Zinc transporter 5	1E-129	Zinc transporter zupT -Medtr3g082050.1	7E-147
Phvulv091018095m	PvZIP12	Chr06	17,174,396	ZIP11_Zinc transporter 11	5.7E-52	Zinc transporter 5 -Medtr2g097580.1	3E-113
Phvulv091002113m	PvZIP13	Chr06	18,954,219	ATZIP6_ZIP metal ion transporter	1.5E-56	Zinc transporter 5 -Medtr5g071990.1	7E-141
Phvulv091007436m	PvZIP14	Chr08	7,634,926	ZIP metal ion transporter family	1.2E-14	Zinc transporter 5 -Medtr7g074060.1	0
Phvulv091022274m	PvZIP15	Chr08	57,181,509	ATZIP6_ZIP metal ion transporter	2E-158	Zinc transporter -Medtr5g071990.1	2E-139
Phvulv091004709m	PvZIP16	Chr08	59,351,699	ZIP1_Zinc transporter 1	3.2E-33	Zinc transporter 6-Medtr3g082050.3	7E-91
Phvulv091010505m	PvZIP17	Chr10	9,817,594	ZIP metal ion transporter family	0	ZIP transporter -Medtr7g074060.1	0
Phvulv091003125m	PvZIP18	Chr11	5,071,268	ZTP29_ZIP metal ion transporter	1.3E-20	Zinc transporter 6, -Medtr4g065640.1	3E-124
Phvulv091030363m	PvZIP19	Chr02	19,642,824	ZIP10_Zinc transporter 10	1E-102	Zinc transporter	3E-174
Phvulv091011372m	PvIRT1	Chr03	49,001,506	IRT1_Iron-regulated transporter 1	4E-126	Zinc transporter	3E-169
Phvulv091011626m	PvIRT2	Chr03	49,013,793	IRT1_Iron-regulated transporter 1	5E-112	Zinc/iron permease-Medtr8g105030.1	6E-129
Phvulv091000876m	PvIRT3	Chr09	12,670,278	ATIRT3_Iron regulated transporter 3	2.2E-68	ZIP transporter - Medtr3g 104400.1	2E-131
Phvulv091000875m	PvIRT4	Chr09	12,670,315	ATIRT3_Iron regulated transporter 3	9.5E-42	Zinc transporter 4—Medtr4g083570.1	3E-174
Phvulv091018638m	bZIP1	Chr05	3,213,447	bZIP23—transcription factor family	7E-49	Basic leucine zipper—Medtr4g073100.1	
Phvulv091015330m	bZIP2	Chr11	3,134,797	bZIP19—transcription factor family	1E-109	Basic leucine zipper—Medtr4g073100.1	5E-57
Phvulv091015414m	bZIP3	Chr11	3,709,270	bZIP44 | basic leucine-zipper 44	2E-52	bZIP transcription factor—Medtr4g070860.1	4E-71

Chromosome and position in base pairs indicate the location of each gene. Their respective homologs in A. thaliana and M. truncatula are shown. The program tBlastn was used to compare the A. thaliana ZIP genes against the bean genome. Homology was based on E–^10^

Gene structure analysis of ZIP genes in *P. vulgaris* revealed that the 23 genes have different intron-exon structures with a wide range of lengths. *PvZIP2, PvZIP6, PvZIP7, PvZIP15*, and *PvIRT1*, are composed of three exons and two introns. *PvZIP3, PvZIP5, PvZIP9, PvZIP10, PvZIP11, PvZIP13, PvZIP16, PvZIP19*, and *PvIRT2* each have four exons and three introns. *PvZIP17* and *PvIRT3* have five exons and four introns. Seven exons were identified in *PvZIP1* and *PvIRT4*. Many exons (10–14) were present in *PvZIP4, PvZIP8, PvZIP12, PvZIP14, and PvZIP18*.

Given the importance of some members of the bZIP gene family in the regulation of ZIP genes and in turn plant Zn homeostasis, their sequences were also characterized in the *P. vulgaris* genome. The common bean genes *bZIP1, bZIP2* and *bZIP3* were 261, 266 and 154 amino acids long, respectively. None of the bZIP genes contained introns. The three amino acids sequences encoding the bZIP genes shared 4.0–38.5% similarity among each other and 15–55.4% similarity with *bZIP19, bZIP23*, and *bZIP24* genes described in *A. thaliana* (Assunção et al., 2010).

### Mapping of PvZIP genes and QTL for seed Fe and Zn concentration

**Figure 3:**
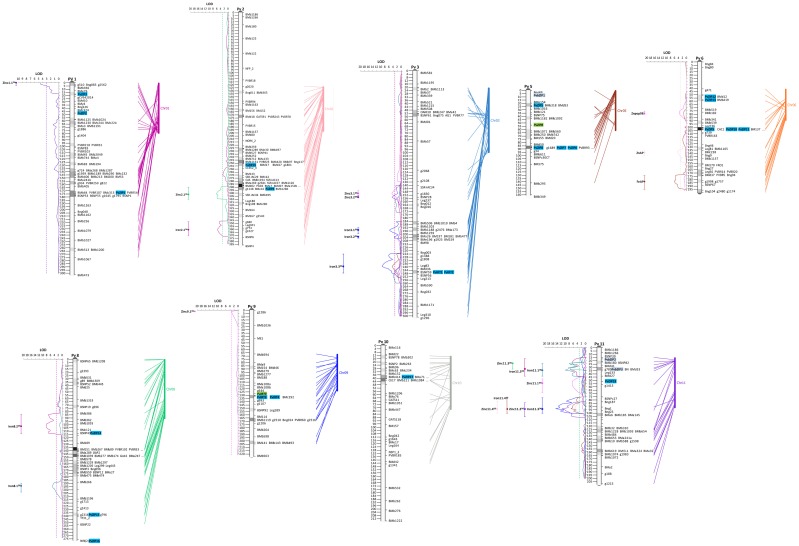
**Genetic mapping, chromosomal location of PvZIP genes and QTLs associated with iron and zinc**. Nineteen ZIP genes and four IRT genes were localized to 9 of 11 chromosomes in *P. vulgaris* on the DOR364 × G19833 genetic map and G19833 sequenced genome. They were aligned for identification of gene position and the coincidence in locations to QTLs with the PvZIP genes. Blue boxes highlight genes mapped *in silico* and green boxes those mapped genetically.

ZIP and bZIP were mapped *in silico* on the DOR364 × G19833 genetic map by aligning ZIP gene sequences and molecular marker sequence in DOR 364 × G19833 against the *P. vulgaris* genome sequence. The results of the *in silico* mapping indicate that ZIP genes are distributed on all *P. vulgaris* chromosomes except 4 and 7. There was a tendency for ZIP genes to cluster together, most notably on chromosome 5 and 6 (Figure 3).

Through *in silico* mapping, *bZIP1* was located on chromosome 5 and *bZIP2* and *bZIP3* were located near each other on chromosome 11. The *bZIP3* gene location was expected based on the position and sequence of the SNP marker g785 which contains the bZIP domain. This marker was described as PvMcCleanNDSU2007_11_g785 (http://cmap.comparative-legumes.org). Selected ZIP genes were also mapped genetically via DNA polymorphisms in the DOR364 × G19833 population using the *P. vulgaris* reference genetic map published by Galeano et al. (2011). This map consists of 499 single copy markers and 2306 cM of coverage (Figure 3). *PvZIP2, PvZIP6*—*PvZIP8, PvZIP13*, and *PvIRT3* mapped to chromosomes 1, 5, 6, and 9, respectively.

Once gene markers were mapped, QTLs for seed Fe and Zn were also identified on this map. These QTLs include previously published data for two sites (Blair et al., 2009) as well as QTL identified in whole and cotyledon seed mineral evaluation from a 2006 planting of the same population in Darien, Colombia. QTL analysis in the 2006 evaluation identified new QTLs for Zn concentration on chromosomes 1 and 2 and also confirmed the QTLs identified by Blair et al. (2009) (Table 3). For seed Fe concentration, 13 QTLs were found on chromosomes 2, 3, 6, 8, and 11. For seed Zn concentration 11 QTLs were found on 1, 2, 3, 6, 9, and 11 (Table 3). ZIP genes mapped on chromosomes 3, 6, 8, and 11 mapped within the regions of QTL for Fe and/or Zn. On chromosome 11 two bZIP genes (with genomic position 3,134,797 and 3,709,270 bp) were mapped *in silico* within the region of two QTLs for seed Fe and one QTL for seed Zn (Figure 3). Two *Pv*IRT genes are present on chromosome 3 (at 49,001,506 and 49,013,793 bp) and three QTLs for seed Fe concentration mapped between the QTLs (Figure 3). Table 3 shows specifically which ZIP genes are located within or nearby QTL for seed mineral concentration.

**Table 3:** **Quantitative trait loci (QTL) for iron and zinc concentration identified with composite interval mapping in the DOR364 × G19833 population**.

**Trait**	**QTL**	**Tissue**	**Environment**	**Chromosome**	**Marker interval**	**Position (cM)**	**ZIP genes nearby^a^**	**Genomic position (bp)**	**LOD**	**R2**	**Adaptive effect**	**Source**
Iron	Iron2.1^DG^^*^	Cotyledon	Darien 2003	2	g680—BSNP6	361.1			5.7	10.6	3.1	G19833
	Iron3.1^DG^	Whole seed	Darien 2003	3	BMb1188—BMb1259	194.8			5.7	11.1	1.8	G19833
	Iron3.2^DG^	Whole seed	Darien 2003	3	g1388—Leg213	226.2	PvIRT1—PvIRT2		9.4	19.7	2.4	DOR364
	Iron3.3^DG^	Whole seed	Darien 2006	3	G1388—BSNP59	239.5	PvIRT1—PvIRT2	49,001,506–49,013,793	4.5	10.5	1.3	DOR364
	Iron 3.4^DG^^*^	Cotyledon	Darien 2003	3	BSNP59	245.5	PvIRT1—PvIRT2		3.3	5.9	2.3	DOR364
	Iron3.5^DG^	Whole seed	Popayan 1998	3	BSNP56—BMb59O	251.1			4.0	9.4	3.4	G19833
	Iron6.1^DG^^*^	Whole seed	Darien 2006	6	PVBR5—Bng104	183.4			6.1	15.3	1.6	G19833
	Iron8.1^DG^	Whole seed	Popayan 1998	8	BMb266—BMb196	192.1			5.0	13.4	3.4	G19833
	Iron8.2^DG^^*^	Cotyledon	Darien 2003	8	BMb386—BSNP43	100.8	PvZIP14	7,634,926	8.2	17.7	4.1	G19833
	Iron11.1^DG^	Whole seed	Popayan 1998	11	BSNP82—BMd27	22.0	Pv bZIP3	3,709,270	9.9	24.5	4.4	G19833
	Iron11.2^DG^	Whole seed	Darien 2003	11	BSNPc27—BMa145	81.4			14.7	34.8	3.0	G19833
	Iron11.3^DG^*	Cotyledon	Darien 2003	11	BSNP39—BMd27	30.8	Pv bZIP2—Pv bZIP3	3,134,797–3,709,270	7.6	15.0	3.6	G19833
	Iron11.4^DG^^*^	Whole seed	Darien 2006	11	BSNPc27—BMa6	79.4			7.2	20.1	1.8	G19833
Zinc	Zinc1.1^DG^^*^	Whole seed	Darien 2003	1	g510—BMb356	0.0	PvZIP3	49,839,431	4.6	8.8	0.6	G19833
	Zinc2.1^DG^^*^	Cotyledon	Darien 2003	2	BMb1286—Leg188	302.5	PvZIP4	33,735,220	6.2	14.1	1.7	DOR364
	Zinc3.1^DG^	Whole seed	Popayan 1998	3	IAC34—BSNP28	150.1			4.6	10.1	1.2	G19833
	Zinc3.2^DG^	Whole seed	Darien 2003	3	g1830—Bng012	154.7			4.5	8.5	0.6	G19833
	Zinc6.1^DG^^*^	Whole seed	Darien 2006	6	BMc238—Bng009	139.3			4.4	13.1	0.7	G19833
	Zinc6.2^DG^	Whole seed	Popayan 1998	6	BMb182	78.8	PvZIP12—PvZIP13	17,174,396–18,954,219	3.6	9.8	1.0	DOR364
	Zinc9.1^DG^	Whole seed	Popayan 1998	9	G1286	0.0			3.2	6.7	1.0	G19833
	Zinc11.1^DG^	Whole seed	Popayan 1998	11	BMd27—BSNPc27	53.4	PvZIP18	5,071,268	6.6	17.7	1.5	G19833
	Zinc11.2^DG^	Whole seed	Darien 2003	11	Bng187—BMa145	92.7			11.5	28.0	1.1	G19833
	Zinc11.3^DG^^*^	Cotyledon	Darien 2003	11	BSNP82—BN	22.0	Pv bZIP2	3,134,797	7.8	17.5	1.8	G19833
	Zinc11.4^DG^^*^	Whole seed	Darien 2006	11	Bng001—BMa6	92.7			5.5	16.0	0.8	G19833

a PvZIP genes and Pv bZIP transcription factor coinciding with QTLs found.

* New QTLs found.

### Expression analysis of *PvZIP* genes

**Figure 4:**
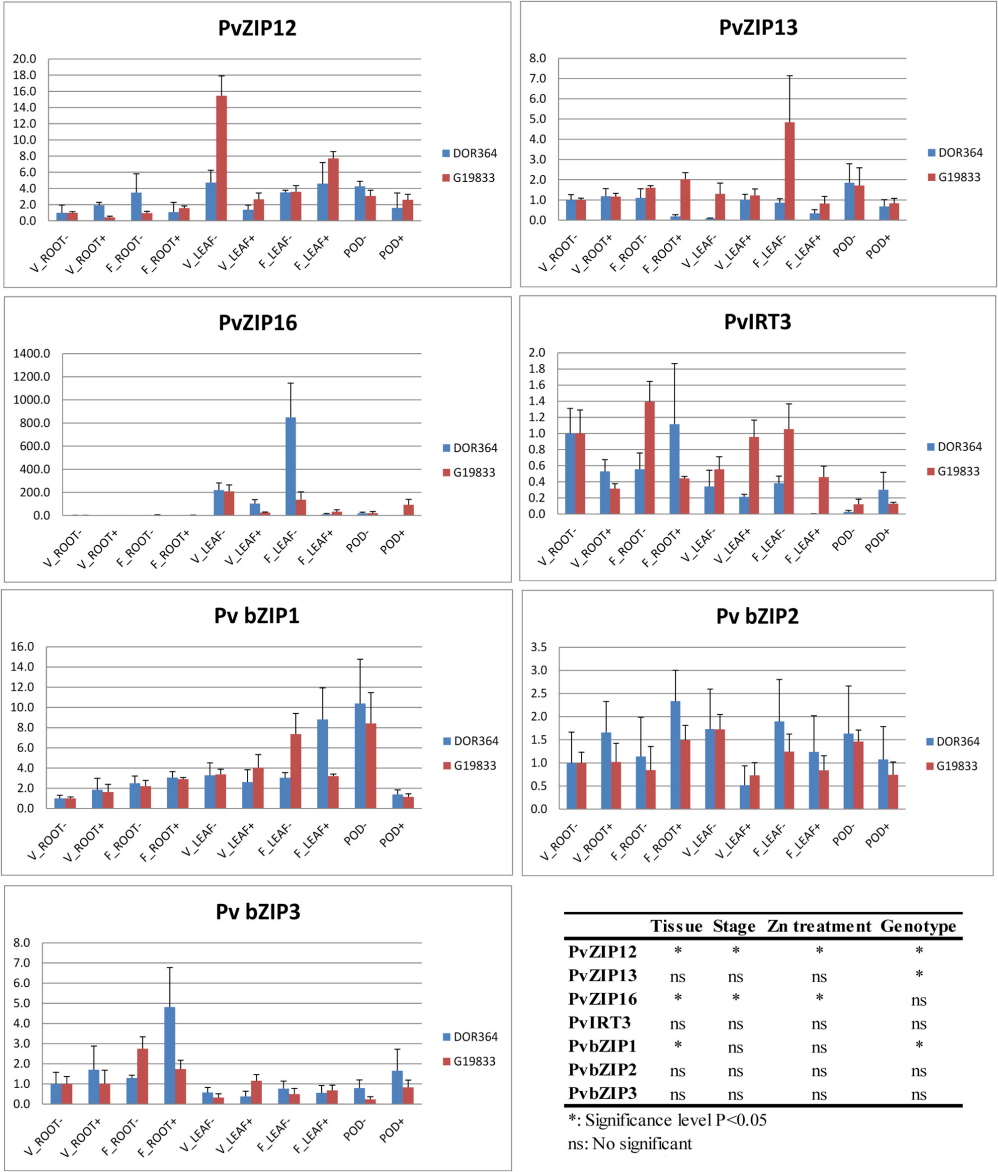
**Relative expression level of PvZIP gene transporters and three bZIP transcription factors in genotypes Dor364 and G19833 in different tissues and two Zn treatment**. (i) roots at vegetative stage (V_ROOT− and V_ROOT+), (ii) roots at flowering stage (F_ROOT− and F_ROOT+); (iii) leaves at vegetative stage (V_LEAF− AND V_LEAF+) stage; (iv) leaves at flowering stage (F_LEAF− and F_LEAF+); and (v) pods (POD− and POD+) of plants under Zn (−) and Zn (+) treatment.

Studies in *Arabidopsis, Glycine, Vitis* and *Medicago* indicate that ZIP genes may be expressed in roots, leaves, and reproductive tissue (Grotz et al., 1998; Lopez-Millan et al., 2004). Many studies so far have focused on expression in roots and shoots (Grotz et al., 1998; Lopez-Millan et al., 2004; Milner et al., 2012). From the perspective of biofortification, it is necessary for a bean plant not only to efficiently take up Zn from the soil, but also transport and accumulate it in vegetative tissue, pods, and ultimately seeds. In order to determine the expression profile of members of ZIP family and their relevance during the development of common bean, relative expression levels were measured by RT qPCR. *PvZIP2, PvZIP7, PvZIP6, PvZIP12, PvZIP13, PvZIP16, PvZIP18*, and *PvIRT3* genes were selected for this analysis based on their location in the genome in relation to presence of QTLs for Zn and Fe in the DOR364 × G19833 population. Three tissue types were analyzed for gene expression in DOR364 and G19833: roots, leaves, and pods. Roots and leaves were collected at two time points, one during vegetative growth, and one during flowering. Pods were sampled 20 days after flowering. Each tissue type was selected from plants with two Zn treatments. At four weeks after planting, DOR364 and G19833 plants in the Zn (−) treatment exhibited some Zn deficiency symptoms such as interveinal chlorosis, bronzing, and shortening of the internode (Brown and Leggett, 1967). The ZIP genes *PvZIP12, PvZIP13, PvZIP16*, and *PvIRT3* were expressed in all tissue analyzed (Figure 4). However, *PvZIP2, PvZIP6, PvZIP7*, and *PvZIP18* were undetectable under RT qPCR in all tissue types. This finding is also supported by pod transcriptome data which also found low to no expression for *PvZIP2, PvZIP6, PvZIP7*, and *PvZIP18* (Astudillo et al., in preparation).

Gene expression of *PvZIP12* and *PvZIP16* was induced upon Zn status in leaf tissue. *PvZIP12* was most highly expressed in leaves at vegetative stage under Zn (−) treatment, especially in G19833 (Figure 4). For *PvZIP13*, G19833 was more highly expressed than DOR364 especially in leaves sampled during flowering. Of each of the ZIP genes studied, *PvZIP16* showed the highest differential expression based on tissue type, stages, and zinc treatment but not between genotypes. It was 139–848-fold more expressed in the leaves than the roots for both genotypes and developmental stages.

### Expression analysis of three transcription factors *bZIP*

RNA from the same samples described above were also used to determine the relative expression of three transcription factors *Pv bZIP1, Pv bZIP2* and *Pv bZIP3*, which are homologous to *Arabidopsis* bZIP genes in the zinc homeostasis network (Table 2). The common bean homologue *bZIP1* was detected in roots, leaves, (at vegetative stages) and pods but expression pattern did not change based on stages and Zn treatment. This gene was more highly expressed in leaf tissue sampled during flowering than vegetative tissue in both G19833 and DOR364. Transcripts of *Pv bZIP2* were detected in roots, leaves, and pods and its expression pattern was not influenced by tissue type, developmental stage or Zn treatment. *Pv bZIP3* was expressed in roots during vegetative and flowering with a barely expression in leaves and pods.

### Tissue Zinc concentration

**Figure 5:**
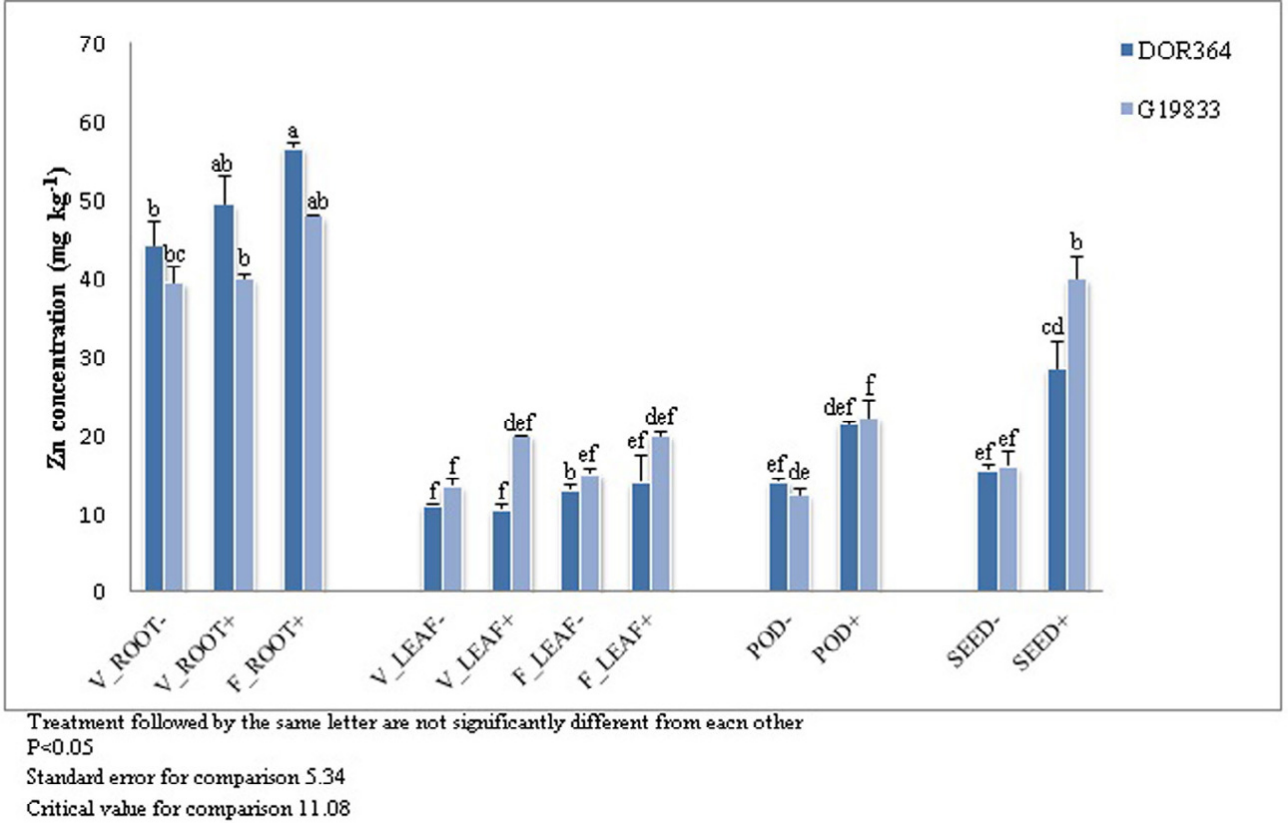
**Zinc concentration in DOR364 and G19833**. Zn concentration (ppm) in (i) roots at vegetative stage (V_ROOT− and V_ROOT+), (ii) roots at flowering stage (F_ROOT+); (iii) leaves at vegetative stage (V_LEAF− AND V_LEAF+) stage; (iv) leaves at flowering stage (F_LEAF− and F_LEAF+); (v) pods (POD− and POD+) and seeds (SEED− and SEED+) of plants under Zn (−) and Zn (+) treatment.

Zn concentration was determined for DOR364 and G19833 for all tissues, developmental stages, and Zn treatments (Figure 5). The highest Zn concentration was observed in roots for both genotypes and no significant differences were observed in leaves. Significant tissue × Zn treatment × genotype interaction was found. Plants grown under the Zn (+) treatment had higher levels of Zn in pods and seeds than those grown under the Zn (−) treatment. Seed Zn levels were 26 and 53% less in the Zn (−) treatment in DOR364 and G19833, respectively. G19833 had higher seed Zn levels that DOR364 under the Zn (+) treatment but not under the Zn (−) treatment (Figure 5).

## Discussion

Common bean is becoming an alternative to dietary supplements as a way to improve human health. ZIP metal transporters are one of the most important gene families for Zn and Fe cellular uptake and translocation in plants (Guerinot, 2000; Chen et al., 2008; Wu et al., 2009; Adams et al., 2012). Identification of ZIP members in *P. vulgaris* and characterization of their expression patterns is useful to increase the understanding of uptake, transportation, and storage of Zn. This study is a unique combination of gene family characterization with physical and genetic mapping and functional expression data that has utility in common bean improvement.

Twenty-three ZIP genes were identified in the *P. vulgaris* genome and genes were annotated and characterized based on similarity to other ZIP family members in *A. thaliana* and *M. truncatula.* According to the total number ZIP family members across species the family origin may be from a common ancestor that has undergone sequence duplication followed by divergence events (D'Ovidio et al., 2004). PvZIP genes clustered on chromosomes 3, 5, 6, and 9 showed high sequence similarity. The close proximity and sequence similarity of many of the ZIP gene family members might suggest of gene duplication followed by diversification (Yang et al., 2009). On the other hand, heterogeneity in structure and expression in each *Pv*ZIP gene suggests diversity in functionality. Four of the eight genes evaluated were not expressed in none of the tissue analyzed. Loss of function in these proteins might be compensated for by duplicate genes (D'Ovidio et al., 2004).

It is important to consider the link between functional variation and gene structural differences among ZIP family members. In many cases, Zn interacts with cysteines and histidines in proteins and may determine the ionic selectivity of ion transporters (Ramesh et al., 2003; Lopez-Millan et al., 2004). The motif of histidine in variable regions between transmembrane domain III and IV in many ZIPs has been postulated to serve as a potential metal ion binding site (Eide et al., 1996; Zhao and Eide, 1996; Grotz et al., 1998). For *Pv*ZIPs identified in this study, all contained this motif except *PvZIP6, PvZIP7*, and *PvZIP18*, interestingly these ZIP genes were also not expressed in all tissue analyzed, suggesting without the motif they are not functional.

In *Arabidopsis*, ZIP genes have been shown to regulate and also contribute to the uptake, transport and accumulation of Zn (Grotz et al., 1998; Weber et al., 2004; Talke et al., 2006; Lin et al., 2009; Milner et al., 2013). Here we used RT-qPCR approach to obtain a picture of ZIP gene transcription in roots and leaves at vegetative and flowering stages, and pods at 20 days after flowering in *P*. *vulgaris*. Some processes such as Zn uptake, have been studied in detail, while others such as remobilization of Zn from vegetative to reproductive tissues are less well understood (Genc et al., 2006). The evaluation of gene expression patterns based on tissue, Zn treatments, and genotype not only provides information on the functionality of the ZIP family genes but also may help explain genotypic differences in seed Zn accumulation. These data indicate differential gene regulation associated with the nutritional requirements and possible mechanism of partitioning of Zn along the plant. According to ZIP genes characterization in *Arabidopsis* approximately half of the genes characterized are induced in response to Zn deficiency (Grotz et al., 1998; Talke et al., 2006).

ZIP gene expression differences in *P. vulgaris* were related to Zn treatments, genotype, and tissue type. Genotypic differences in Zn translocation capacity in different organs may be an important factor in Zn accumulation in seeds (Hacisalihoglu et al., 2004). Observed differences between genotypes could also be due to genetic differences and diversity among Andean and Mesoamerican gene pools (Blair et al., 2009). Similarly to previous studies, G19833 had higher seed Zn level than DOR364 (Blair et al., 2009). However DOR364 had higher Zn in its roots as compared to G19833 suggesting that G19833 can translocate more Zn from roots to seeds.

Zinc plays a specific role in fertilization and pollen grains contain very high concentrations of Zn (Fageria et al., 2011). At flowering most of the Zn taken up is incorporated into the developed seed (Jiang et al., 2008) so genes highly expressed at flowering and in pods such as *PvZIP12, PvZIP16*, and *bZIP1* could be directly related to Zn remobilization to seeds.

Although leaves are known as the major source of remobilized micronutrients in common bean (Sekara et al., 2005) in rice stems are the major source of Zn in the seed (Waters and Sankaran, 2011). With this study it was not possible to determine how much and the source of Zn remobilization. Future studies with radio labeled Zn would be warranted to asses Zn remobilization.

Based on the relative expression values established via RT-qPCR, the high Zn concentration in roots did not reflect expression values for the ZIP genes evaluated in this tissue. In *Arabidopsis* at least 10 different members of the ZIP family play a role in zinc uptake in roots, including ZIP1, 2, 3, 4, 5, 9, 10, 11, 12, and IRT3 (Van De Mortel et al., 2006). We evaluated four of their respective homologous in *P. vulgaris* and found that they were only weakly expressed in roots.

The DOR364 × G19833 RILs mapping population consists of 86 individuals, which are adequate for identifying QTL with moderately large effects based on QTLs previously detected (Blair et al., 2009, 2011; Galeano et al., 2011). *In silico* mapping of ZIP genes was a successful strategy to locate *Pv*ZIP genes aligned with QTL for seed Fe and Zn in the bean genome. QTL analysis was carried out in the current reference bean map (Galeano et al., 2011). It is worth noting that on chromosomes 2 and 6 where *PvZIP4* and *PvZIP12* and *PvZIP13* are located, there are QTL for seed Zn concentration. For Fe, the IRT genes are considered to be the main transporters for high-affinity iron uptake in roots in *Arabidopsis* (Connolly et al., 2002; Henriques et al., 2002; Lin et al., 2009). In this study, *PvIRT1* and *PvIRT2* were located on chromosome 3 within an important QTL region associated with seed Fe concentration. The *Pv bZIP2* and *Pv bZIP3* genes were located on chromosome 11 and aligned with the most important QTL for Fe and Zn reported in *P. vulgaris*. There are no obvious genotypic differences in expression of these genes in G19833 and DOR 364, however. The QTL in this region has been found in at least three mapping populations, including Mesoamerican and Andean intra and inter genepool crosses (Blair et al., 2009, 2010a,b, 2011). The bZIP transcription factors analyzed correspond to genes in *Arabidopsis* responsible for response and adaptation to low Zn supply. In general, *Pv*ZIP, *Pv*IRT, and *Pv* bZIPs co-localization with QTLs for Fe and Zn levels suggest that their function is important in Fe and Zn homeostasis in *P. vulgaris*. In *Arabidopsis*, the bZIP transcription factors that interacted with ZIP genes were found directly upstream of the ZIP genes (Assunção et al., 2010). In the case of *P. vulgaris* none of the bZIP genes were adjacent to ZIP genes.

This study is the first to characterize the ZIP gene family, report the expression profile in various tissues with two genotypes and fertilization treatments. It provides evidence of the relationship among level of transcripts and QTLs in dry bean seed as was identified in *PvZIP12* gene. This contribution will be particularly useful for advancing bean breeding programs. The use of such gene marker encoding proteins associated with transport and accumulation of Zn and Fe could increase the efficiency and accuracy in the selection of breeding materials for biofortification.

### Conflict of interest statement

The authors declare that the research was conducted in the absence of any commercial or financial relationships that could be construed as a potential conflict of interest.
